# The EU Child Cohort Network’s core data: establishing a set of findable, accessible, interoperable and re-usable (FAIR) variables

**DOI:** 10.1007/s10654-021-00733-9

**Published:** 2021-04-21

**Authors:** Angela Pinot de Moira, Sido Haakma, Katrine Strandberg-Larsen, Esther van Enckevort, Marjolein Kooijman, Tim Cadman, Marloes Cardol, Eva Corpeleijn, Sarah Crozier, Liesbeth Duijts, Ahmed Elhakeem, Johan G. Eriksson, Janine F. Felix, Sílvia Fernández-Barrés, Rachel E. Foong, Anne Forhan, Veit Grote, Kathrin Guerlich, Barbara Heude, Rae-Chi Huang, Marjo-Riitta Järvelin, Anne Cathrine Jørgensen, Tuija M. Mikkola, Johanna L. T. Nader, Marie Pedersen, Maja Popovic, Nina Rautio, Lorenzo Richiardi, Justiina Ronkainen, Theano Roumeliotaki, Theodosia Salika, Sylvain Sebert, Johan L. Vinther, Ellis Voerman, Martine Vrijheid, John Wright, Tiffany C. Yang, Faryal Zariouh, Marie-Aline Charles, Hazel Inskip, Vincent W. V. Jaddoe, Morris A. Swertz, Anne-Marie Nybo Andersen, for the LifeCycle Project Group

**Affiliations:** 1 Section for Epidemiology, Department of Public Health, University of Copenhagen, Copenhagen, Denmark; 2 Genomics Coordination Center, University of Groningen, University Medical Center Groningen, Groningen, The Netherlands; 3 Department of Pediatrics, Erasmus MC, University Medical Center Rotterdam, PO Box 2040, 3000 CA Rotterdam, The Netherlands; 4 Generation R Study Group, Erasmus MC, University Medical Center Rotterdam, PO Box 2040, 3000 CA Rotterdam, The Netherlands; 5 Population Health Science, Bristol Medical School, Bristol, BS8 2BN UK; 6 MRC Integrative Epidemiology Unit, University of Bristol, Bristol, BS8 2PS UK; 7 Department of Epidemiology, University of Groningen, University Medical Center Groningen, Groningen, The Netherlands; 8 MRC Lifecourse Epidemiology Unit, University of Southampton, Southampton General Hospital, Southampton, UK; 9 NIHR Applied Research Collaboration Wessex, Southampton Science Park, Innovation Centre, 2 Venture Road, Chilworth, Southampton, SO16 7NP UK; 10 Department of General Practice and Primary Health Care, University of Helsinki and Helsinki University Hospital, Helsinki, Finland; 11 Folkhälsan Research Center, Helsinki, Finland; 12 Obstetrics and Gynecology, Yong Loo Lin School of Medicine, National University of Singapore and National University Health System, Singapore, Singapore; 13 Singapore Institute for Clinical Sciences (SICS), Agency for Science and Technology (A*STAR), Singapore, Singapore; 14 ISGlobal, Barcelona, Spain; 15 Universitat Pompeu Fabra (UPF), Barcelona, Spain; 16 CIBER Epidemiología y Salud Pública (CIBERESP), Barcelona, Spain; 17 Telethon Kids Institute, Perth, WA Australia; 18 School of Physiotherapy and Exercise Science, Curtin University, Perth, WA Australia; 19 Université de Paris, Centre for Research in Epidemiology and Statistics (CRESS), INSERM, INRAE, Paris, France; 20 Division of Metabolic and Nutritional Medicine, Department of Pediatrics, Dr. von Hauner Children’s Hospital, LMU University Hospital Munich, Munich, Germany; 21 Faculty of Medicine, Center for Life-Course Health Research, University of Oulu, P.O. Box 5000, 90014 Oulu, Finland; 22 Department of Epidemiology and Biostatistics, School of Public Health, Imperial College London, London, UK; 23 Clinicum, Faculty of Medicine, University of Helsinki, Helsinki, Finland; 24 Department of Genetics and Bioinformatics, Division of Health Data and Digitalisation, Norwegian Institute of Public Health, Oslo, Norway; 25 Cancer Epidemiology Unit, Department of Medical Sciences, University of Turin, Turin, Italy; 26 Department of Social Medicine, Faculty of Medicine, University of Crete, Heraklion, Crete, Greece; 27 Bradford Institute for Health Research, Bradford Teaching Hospitals NHS Foundation Trust, Bradford, UK; 28 ELFE Joint Unit, French Institute for Demographic Studies (Ined), French Institute for Medical Research and Health (INSERM), French Blood Agency, Aubervilliers, France; 29 NIHR Southampton Biomedical Research Centre, University of Southampton and University Hospital Southampton NHS Foundation Trust, Southampton, UK; 30 Department of Genetics, University of Groningen, University Medical Center Groningen, Groningen, The Netherlands

**Keywords:** Birth cohort, Cross-cohort collaboration, Lifecourse epidemiology, Data harmonisation, FAIR (findable, accessible, interoperable and reusable) principles

## Abstract

The Horizon2020 LifeCycle Project is a cross-cohort collaboration which brings together data from multiple birth cohorts from across Europe and Australia to facilitate studies on the influence of early-life exposures on later health outcomes. A major product of this collaboration has been the establishment of a FAIR (findable, accessible, interoperable and reusable) data resource known as the EU Child Cohort Network. Here we focus on the EU Child Cohort Network’s core variables. These are a set of basic variables, derivable by the majority of participating cohorts and frequently used as covariates or exposures in lifecourse research. First, we describe the process by which the list of core variables was established. Second, we explain the protocol according to which these variables were harmonised in order to make them interoperable. Third, we describe the catalogue developed to ensure that the network’s data are findable and reusable. Finally, we describe the core data, including the proportion of variables harmonised by each cohort and the number of children for whom harmonised core data are available. EU Child Cohort Network data will be analysed using a federated analysis platform, removing the need to physically transfer data and thus making the data more accessible to researchers. The network will add value to participating cohorts by increasing statistical power and exposure heterogeneity, as well as facilitating cross-cohort comparisons, cross-validation and replication. Our aim is to motivate other cohorts to join the network and encourage the use of the EU Child Cohort Network by the wider research community.

**Supplementary Information:**

The online version contains supplementary material available at 10.1007/s10654-021-00733-9.

## Introduction

Non-communicable diseases (NCDs) such as cardiovascular disease, cancer, chronic respiratory disease and diabetes represent a major global health challenge and are the leading cause of death worldwide. Of the 56.9 million deaths that occurred in 2016, 40.5 million (71%) were from NCDs [1]; this number is estimated to rise to 52 million by 2030 [2]. To address the growing economic and health burden that NCDs represent, the United Nations’ Sustainable Development Goal (SDG) target 3.4 aims to reduce premature mortality due to NCDs by one third by 2030 through prevention, treatment and promotion of mental health and wellbeing [1].

Early-life offers an important window of opportunity for achieving this target. Evidence strongly suggests that environmental conditions and exposures during intrauterine and early postnatal life can influence anatomical, physiological and biochemical processes and, in so doing, impact future health [3]. Longitudinal pregnancy and child cohort studies provide a means of investigating this phenomenon, including how early-life exposures influence health trajectories, and identifying potential early-life interventions to improve health outcomes [4]. However, such studies are expensive to establish and maintain, which often prohibits the large-scale studies required to investigate rare outcomes or exposures, or conduct more advanced statistical analyses to investigate, for example, causality or lifecourse health trajectories.

Cross-cohort collaborations offer a cost-effective approach to increase the statistical power of such analyses. They also provide other benefits such as increased exposure heterogeneity, facilitated cross-cohort comparisons, the ability to cross-validate, replicate and establish the generalisability of findings, and the opportunity to share expertise and knowledge. In recent years, a number of such collaborations have been successfully established, for example the CHICOS (www.chicosproject.eu), BioSHARE [5], HELIX [6] (www.projecthelix.eu), PACE [7], EGG/EAGLE [8], ESCAPE [9] (www.escapeproject.eu) and Enrieco [10] (www.enrieco.org) projects, which have led to the identification of a number of associations that may have otherwise gone unobserved [11–28]. More recently, in 2017, building on expertise gained from these collaborations, the Horizon 2020-funded LifeCycle project was established [29] (www.lifecycle-project.eu).

LifeCycle aims to facilitate the utilisation of data from mainly European, but also some non-European, cohort studies for research. It has a particular focus on preconception, fetal and early childhood exposures and their influence on cardio-metabolic, respiratory and mental health trajectories. To achieve its aim, LifeCycle has established the EU Child Cohort Network, a sustainable data resource and infrastructure which is built around making each participating cohort’s data findable, accessible, interoperable and reusable (FAIR) [30]. The network currently holds data on approximately 250,000 children and their parents from an initial 16 European and one Australian cohort.

An overview of the EU Child Cohort Network, including the data management and governance structure on which the network is based, plus its primary research themes, was provided by Jaddoe et al. in a previous edition of this journal [29]. Here we provide a detailed description of the EU Child Cohort Network’s core variables, which are a set of basic variables, derivable by the majority of participating cohorts and required for most analyses in lifecourse research. We describe firstly the process by which the list of core variables was established; secondly the protocol developed to harmonise these core data, which defines the harmonisation process adopted generally within LifeCycle; thirdly the catalogue developed to ensure that all EU Child Cohort Network data are both findable and reusable; finally the core data themselves, including the variables harmonised by each cohort and the total number of children with harmonised data. Our aims are to: (1) enable an accurate assessment of the quality and validity of the harmonised core data through transparency of our methods; (2) motivate other cohorts to contribute to the network; (3) encourage the use of the EU Child Cohort Network’s data by the wider scientific community.

## Methods

### Participating cohorts

An overview of the 17 cohorts that established the EU Child Cohort Network is provided in Table 1. Further details of each cohort can be found in Jaddoe et al. [29], the EU Child Cohort Network Variable Catalogue (http://catalogue.lifecycle-project.eu) and each cohort’s profile paper [31–49]. The network is open for other cohorts to join, provided they meet the following criteria: (1) commenced before or during pregnancy or in infancy; (2) plan to follow-up or already have followed-up the cohort throughout childhood; (3) are willing to harmonise data and make them available to researchers using the network. Cohorts can join the network by contacting the coordinating centre (lifecycle@erasmusmc.nl). Similarly, proposals for research based on EU Child Cohort Network data can be put forward by both LifeCycle partners and external researchers by also contacting the coordinating centre (lifecycle@erasmusmc.nl). Proposals for research may be based on all EU Child Cohort Network cohorts or a subset of cohorts with available data; they may also include requests for further data harmonisation, which can likewise be restricted to a subset of cohorts with data.

**Table 1:** Pregnancy and child cohorts contributing data to the EU Child Cohort Network as of June 2020

Cohort (full name)	Country	Recruitment	Enrolment period	Age at last follow-up (y)	N^a^
ALSPAC (Avon Longitudinal Study of Parents & Children)	UK	1991–1992	Pregnancy	25	10,742
BiB (Born in Bradford)	UK	2007–2011	Pregnancy	9	12,397
CHOP (The EU Childhood Obesity Programme)	Germany, Belgium, Italy, Spain and Poland	2002–2004	Birth	11	1280
DNBC (Danish National Birth Cohort)	Denmark	1996–2002	Pregnancy	18	72,157
EDEN (Study on the pre- & early postnatal determinants of child health & development)	France	2003–2005	Pregnancy	8	1676
ELFE (Etude Longitudinale Francaise depuis l’Enfance)	France	2011	Birth	7	10,825
GECKO (Groningen Expert Center for Kids with Obesity Drenthe Cohort)	The Netherlands	2006–2007	Pregnancy	10	2682
Gen R (Generation R)	The Netherlands	2002–2006	Pregnancy	17	8534
HBCS (Helsinki Birth Cohort Study)	Finland	1934–1944	Birth	76	13,343
INMA (INMA-Infancia y Medio Ambiente (Environment and Childhood Project))	Spain	1997–2008	Pregnancy	18	1900
MoBa (Norwegian Mother, Father and Child Cohort Study)	Norway	1999–2008	Pregnancy	14	76,569
NFBC1966 (Northern Finland Birth Cohort 1966)	Finland	1966	Pregnancy	46–48	7810
NFBC1986 (Northern Finland Birth Cohort 1986)	Finland	1985–1986	Pregnancy	33–35	8372
NINFEA (Nascita e INFanzia: gli Effetti dell’Ambiente)	Italy	2005–2016	Pregnancy	13	6018
Raine (The Raine Study)	Australia	1989–1992	Pregnancy	26	2491
Rhea (Mother Child Cohort in Crete)	Greece	2007–2008	Pregnancy	7	967
SWS (Southampton Women’s Survey)	UK	1998–2007	Preconception	9	2921

^a^Number of children from the cohort contributing data to the EU Child Cohort Network and with all three of the following variables harmonised: (1) birth weight, (2) sex, (3) at least one height or weight measurement taken at ≥ 1 year

**Table 2:** Child-related characteristics of cohorts contributing data to the EU Child Cohort Network

Cohort	N^a^	Female, n (%)	GA (weeks), mean (SD)	Birth weight (g), mean (SD)	SGA^b^, n (%)	LGA^c^, n (%)	Ever breastfed, n (%)
ALSPAC	10,742	5313 (49.5)	40.0 (1.9)	3408 (555)	644 (6.0)	1015 (9.5)	7213 (75.8)
BiB	12,397	5980 (48.2)	39.5 (1.8)	3212 (557)	1385 (11.2)	562 (4.5)	3228 (78.7)
CHOP	1280	659 (51.5)	40.4 (1.2)	3297 (351)	28 (2.2)	34 (2.7)	901 (70.4)
DNBC	72,157	35,464 (49.1)	39.9 (1.8)	3565 (582)	2281 (3.2)	10,046 (14.0)	55,214 (98.3)
EDEN	1676	802 (47.9)	39.7 (1.7)	3283 (506)	118 (7.0)	60 (3.6)	1230 (73.4)
ELFE	10,825	5277 (48.7)	39.6 (1.5)	3322 (488)	644 (6.0)	535 (5.0)	7858 (74.8)
GECKO	2682	1332 (49.7)	39.8 (1.6)	3542 (548)	87 (3.3)	357 (13.4)	1938 (79.4)
Gen R	8534	4229 (49.6)	40.3 (1.9)	3400 (576)	615 (7.4)	541 (6.5)	6013 (91.8)
HBCS	13,343	6369 (47.7)	39.8 (1.8)	3407 (479)	NA	NA	11,110 (99.6)
INMA	1900	923 (48.6)	39.9 (1.6)	3263 (467)	139 (7.3)	70 (3.7)	1648 (88.6)
MoBa	76,569	37,390 (48.8)	39.8 (1.9)	3576 (578)	2725 (3.6)	7377 (9.6)	71,768 (93.7)
NFBC1966	7810	3628 (46.5)	40.5 (1.9)	3491 (530)	378 (5.3)	703 (9.9)	4550 (86.0)
NFBC1986	8372	4112 (49.1)	39.8 (1.7)	3560 (546)	259 (3.1)	1186 (14.2)	NA
NINFEA	6018	2951 (49.0)	39.7 (1.7)	3238 (493)	471 (7.9)	200 (3.3)	5502 (92.1)
Raine	2491	1218 (48.9)	39.1 (2.3)	3299 (602)	142 (7.0)	146 (7.2)	2082 (89.7)
Rhea	967	459 (47.5)	38.7 (1.5)	3183 (455)	56 (5.9)	51 (5.3)	805 (86.5)
SWS	2921	1411 (48.3)	39.7 (1.8)	3441 (547)	126 (4.3)	259 (8.9)	2376 (82.5)

Values are mean (standard deviation) or n (valid percent)

*GA* gestational age at birth, *SGA* small for gestational age, *LGA* large for gestational age, *NA* data not available

^a^Number of children from the cohort contributing data to the EU Child Cohort Network and with all three of the following variables harmonised: i) birth weight, ii) sex, iii) at least one height or weight measurement taken at ≥ 1 year

^b^Birth weight ≤ 5th percentile for gestational age (in completed weeks) using the WHO fetal growth charts [52] as the growth standard

^c^Birth weight ≥ 95th percentile for gestational age (in completed weeks) using the WHO fetal growth charts [52] as the growth standard

**Table 3:** Mother-related characteristics of cohorts contributing data to the EU Child Cohort Network

Cohort	N^a^	Maternal age at birth (y), mean (SD)	Education level, n (%)			Ethnicity, n (%)			Multiparous, n (%)	Smoked in pregnancy, n (%)
			High	Medium	Low	White	Black, Asian or minority ethnic	Mixed		
ALSPAC	10,742	29.2 (4.6)	1444 (14.2)	6954 (68.6)	1741 (17.2)	9874 (98.3)	169 (1.7)	–	5629 (54.8)	2468 (26.0)
BiB	12,397	27.6 (5.6)	2534 (26.8)	1502 (15.9)	5420 (57.3)	4290 (41.8)	5783 (56.3)	200 (1.9)	7259 (60.8)	1659 (16.2)
CHOP	1280	30.2 (5.0)	336 (26.3)	640 (50.2)	300 (23.5)	1232 (96.4)	46 (3.6)	–	652 (51.0)	416 (32.6)
DNBC	72,157	30.1 (4.2)	33,700 (52.3)	14,067 (21.8)	16,655 (25.9)	NA	NA	NA	37,964 (52.6)	17,580 (24.7)
EDEN	1676	29.7 (4.8)	938 (56.2)	636 (38.1)	94 (5.6)	1437 (99.1)	7 (0.5)	6 (0.4)	911 (54.5)	413 (24.7)
ELFE	10,825	30.8 (4.7)	7240 (66.9)	3063 (28.3)	521 (4.8)	8706 (83.9)	963 (9.3)	705 (6.8)	5673 (53.0)	1779 (16.6)
GECKO	2682	30.7 (4.4)	900 (35.9)	724 (28.9)	885 (35.3)	2400 (95.5)	70 (2.8)	43 (1.7)	1591 (59.9)	411 (15.4)
Gen R	8534	30.7 (5.2)	3448 (45.3)	3380 (44.4)	778 (10.2)	4606 (57.1)	2665 (33.0)	799 (9.9)	3691 (44.8)	1888 (25.9)
HBCS	13,343	28.4 (5.4)	NA	NA	NA	NA	NA	NA	6861 (51.4)	NA
INMA	1900	31.8 (4.2)	661 (35.2)	768 (40.9)	449 (23.9)	1802 (95.7)	80 (4.3)	–	810 (44.5)	588 (31.4)
MoBa	76,569	30.4 (4.4)	48,804 (67.5)	22,166 (30.6)	1354 (1.9)	NA	NA	NA	39,262 (51.7)	6194 (8.1)
NFBC1966	7810	28.1 (6.7)	254 (3.3)	1033 (13.5)	6387 (83.2)	NA	NA	NA	5387 (69.1)	1569 (20.7)
NFBC1986	8372	27.8 (5.5)	1735 (23.7)	2744 (37.4)	2856 (38.9)	NA	NA	NA	5499 (65.9)	1975 (23.7)
NINFEA	6018	33.2 (4.2)	3799 (63.6)	1923 (32.2)	253 (4.2)	NA	NA	NA	1548 (27.0)	453 (7.6)
Raine	2491	27.9 (5.8)	465 (20.1)	633 (27.3)	1221 (52.7)	2175 (89.2)	264 (10.8)	–	1275 (52.3)	666 (27.3)
Rhea	967	29.7 (4.9)	304 (32.1)	481 (50.7)	163 (17.2)	926 (99.8)	2 (0.2)	–	524 (54.9)	290 (33.1)
SWS	2921	30.2 (3.8)	837 (28.7)	1730 (59.2)	345 (11.8)	2799 (95.8)	105 (3.6)	16 (0.5)	1409 (48.3)	428 (15.4)

Values are mean (standard deviation) or n (valid percent)

^a^Number of children from the cohort contributing data to the EU Child Cohort Network and with all three of the following variables harmonised: (1) birth weight, (2) sex, (3) at least one height or weight measurement taken at ≥ 1 year. Mothers who contributed more than one child to a cohort are counted more than once in the table

### Harmonisation

**Fig. 1:**
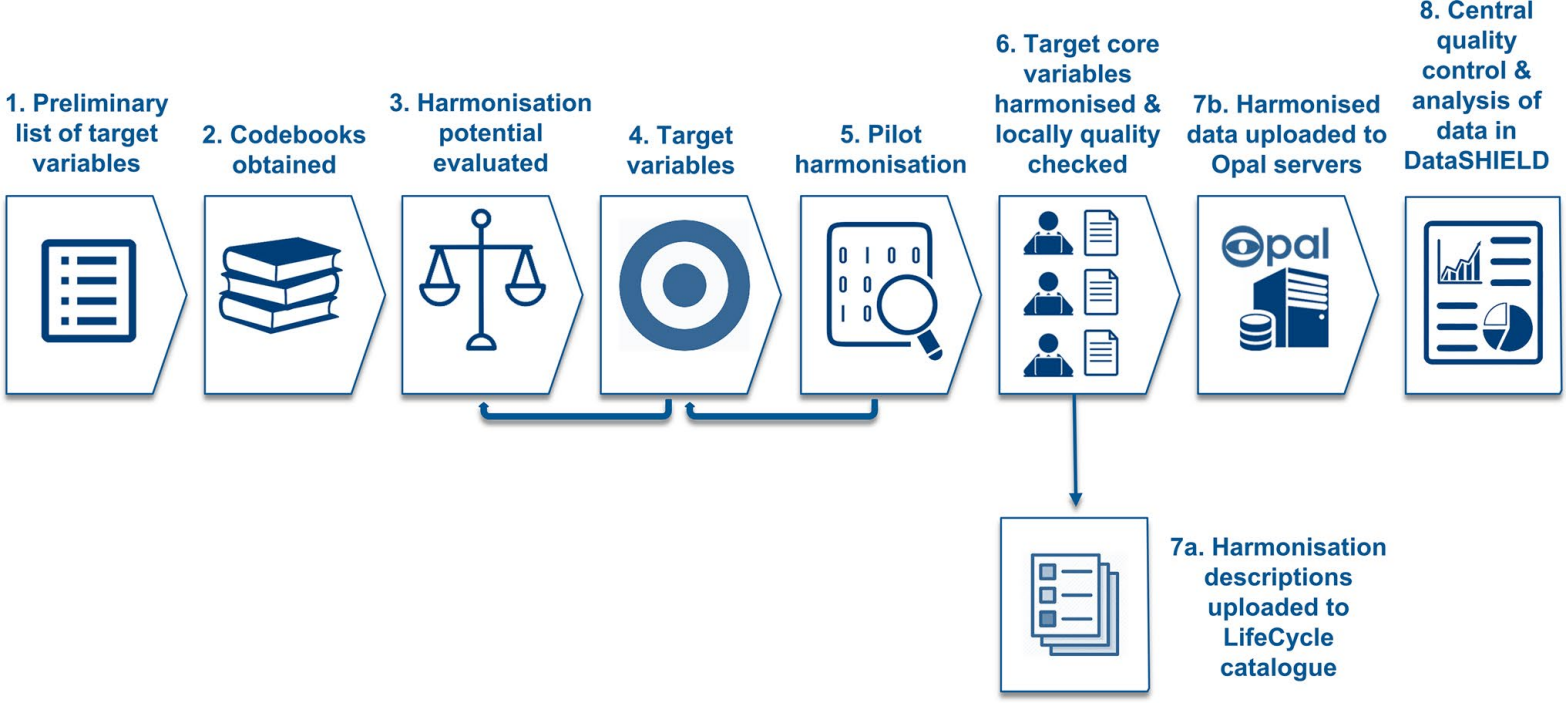
The process adopted in LifeCycle to establish and harmonise the core variables for the EU Child Cohort Network

The EU Child Cohort Network’s core variables are a set of basic, predominantly “lowest common denominator” variables, derivable by the majority of participating cohorts and frequently needed as covariates or exposures in lifecourse research. The process adopted in LifeCycle to establish and harmonise these core variables for the EU Child Cohort Network can be broken down into eight steps; an overview of these steps is displayed in Fig. 1. A glossary of the key elements and concepts described in this paper is also provided in Box 4.

**Box 1:** A glossary of the key elements and concepts in LifeCycle

Term	Definition
Complete harmonisation	The ability to derive the variable as described in the harmonization manual, both in definition and format
Data harmonisation	The process of creating a common dataset from disparate datasets
DataSHIELD	An infrastructure and series of R packages that enables the remote and non-disclosive analysis of individual participant data
EU Child Cohort Network	A network bringing together existing data from more than 250,000 European and Australian children and their parents
Federated data analysis	Centralised analysis of individual participant data where data are stored on local servers and do not leave the host institution
Harmonisation manual	A manual containing a list of target variables together with instructions for their harmonisation
Impossible harmonisation	The complete inability to derive the variable due to no or limited information
Horizon2020 LifeCycle Project	A collaboration between scientists from more than 17 existing pregnancy and child cohort studies
EU Child Cohort Network Variable Catalogue	An online catalogue providing an overview of available data in the EU Child Cohort Network, including details of how data have been created (http://catalogue.lifecycle-project.eu)
LifeCycle core variables	A set of basic variables, derivable by the majority of cohorts participating in LifeCycle and frequently required in lifecourse analyses
Opal	A data warehouse that is integrated with R and the DataSHIELD platform, allowing the analysis of data without the physical sharing or disclosing of individual participant data
Partial harmonisation	The ability to derive the variable as described but with some loss of information

#### Step 1: establishing a preliminary list of target core variables

LifeCycle partners with expertise in a wide range of fields including lifecourse epidemiology, public health, environmental epidemiology, biology, statistics, paediatrics, obstetrics, economics, demography, epigenomics and data science, met in a dedicated workshop (June 2017) to identify a preliminary list of core early-life stressors and exposures related to cardio-metabolic, respiratory and mental health outcomes using a consensus approach. This initial list was then further modified by drawing on experiences from other previous collaborative efforts such as MOBAND [50] and CHICOS (www.chicosproject.eu), and through consulting the literature and experts in the field, before being circulated amongst LifeCycle partners for further comment.

#### Steps 2, 3 & 4: collating codebooks, evaluating the harmonisation potential of each variable and finalising a list of target core variables

All cohorts participating in LifeCycle were requested to provide the coordinating team with cohort metadata (codebooks, questionnaires, instrument documentation, etc.). From these, the potential for each cohort to derive each target variable was established. The core variable list was then adapted in an iterative manner to achieve a balance between precision and inclusivity, ensuring a maximum number of cohorts could contribute data for numerous variables while maintaining data validity. Where possible, international standards and classification schemes were applied. For example, the International Standard Classification of Occupation 1988 1-digit codes [51] were used to categorise parental occupation; the International Standard Classification of Education 97/2011 schemes [52, 53] were used to classify parental education; the WHO fetal growth charts [54] were used to establish size-for-gestational-age; the EUROCAT guide was used for classifying congenital anomalies. For some key exposures such as maternal smoking, breastfeeding, childcare attendance and gestational age, several variables were included, with some variables capturing more information but at the cost of fewer cohorts being able to derive the variables. Repeated measures were also included, to capture the dynamic, longitudinal nature of many variables.

#### Step 5: pilot harmonisation

Data harmonisation was staggered across cohorts. First, an initial pilot harmonisation was conducted among four cohorts covering the majority of target core variables (the Danish National Birth Cohort, the EDEN mother-child cohort, the Generation R study and the Southampton Women’s Survey). This enabled any potential issues in the core variable list to be identified and rectified. During the pilot harmonisation, the core variable list was revised iteratively through electronic communication, a workshop and a final teleconference.

#### Step 6: data harmonisation and local quality control

Harmonisation for the EU Child Cohort Network was carried out locally by each participating cohort. This avoided any transfer of data but carried the risk of harmonisation protocols being interpreted differently by different cohorts. To limit this possibility, a detailed harmonisation manual was drawn up by the coordinating team, and supervision and feedback was maintained between the coordinating centre and each of the cohorts. The harmonisation manual is available to download from the LifeCycle website (https://lifecycle-project.eu); it includes: (1) a final, annotated list of core variables, which, for each variable, includes: a variable name, a precise definition, a label, units, data type, permissible values and guidelines for what constitutes partial versus complete harmonisation (see Box 4 for definitions of partial vs. complete harmonisation); (2) relevant scale conversions; (3) relevant reference tables (e.g. WHO fetal growth charts, the EUROCAT guide for classifying congenital anomalies etc.). The harmonisation manual was circulated to cohorts in May 2018 and harmonisation of core variables by all cohorts was completed by May 2020. The duration of time that it took a cohort to harmonise all core variables ranged from three to eight months.

Once data were harmonised, each cohort was provided with detailed quality control instructions and scripts to check: (1) that variables matched the descriptions provided in the core variable list (name, datatype, values); (2) for outliers or improbable values; (3) for inconsistencies between non-repeated measures (e.g. all mothers coded as not smoking during pregnancy were also coded as smoking zero cigarettes during pregnancy); (4) for inconsistencies between repeated measures (e.g. children reducing height over time). Any inconsistencies identified were investigated on a cases-by-case basis to establish which values were legitimate and which were errors, also in light of the other data available.

#### Step 7a: uploading harmonisation descriptions to the EU Child Cohort Network variable catalogue

**Fig. 2:**
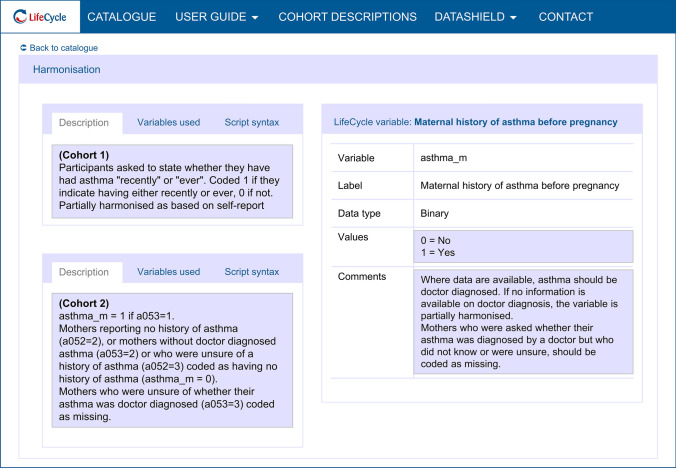
An illustration of the EU Child Cohort Network Variable Catalogue displaying the LifeCycle variable “maternal history of asthma before pregnancy”. Displayed is a description of the target EU Child Cohort Network variable and how the variable was harmonised in two separate cohorts. Note: descriptions from two separate cohorts are displayed on the same page for illustrative purposes only

To facilitate the utilisation of EU Child Cohort Network data for research, and ensure the complete and accurate documentation of harmonisation, an online catalogue of EU Child Cohort Network variables was developed using the Molgenis platform [55] (http://catalogue.lifecycle-project.eu). This open source, searchable catalogue includes detailed descriptions of each variable included in the EU Child Cohort Network (variable name, data type, values, unit and description), as well as details of which cohorts have harmonised each variable, whether that harmonisation was complete or partial, an explanation of how the variable was harmonised, plus the syntax and descriptions of the source variables used by each cohort to derive the variable (Fig. 2). For the core variables, documentation of harmonisation was conducted by each cohort and uploaded to the catalogue after harmonisation was complete.

**Fig. 3:**
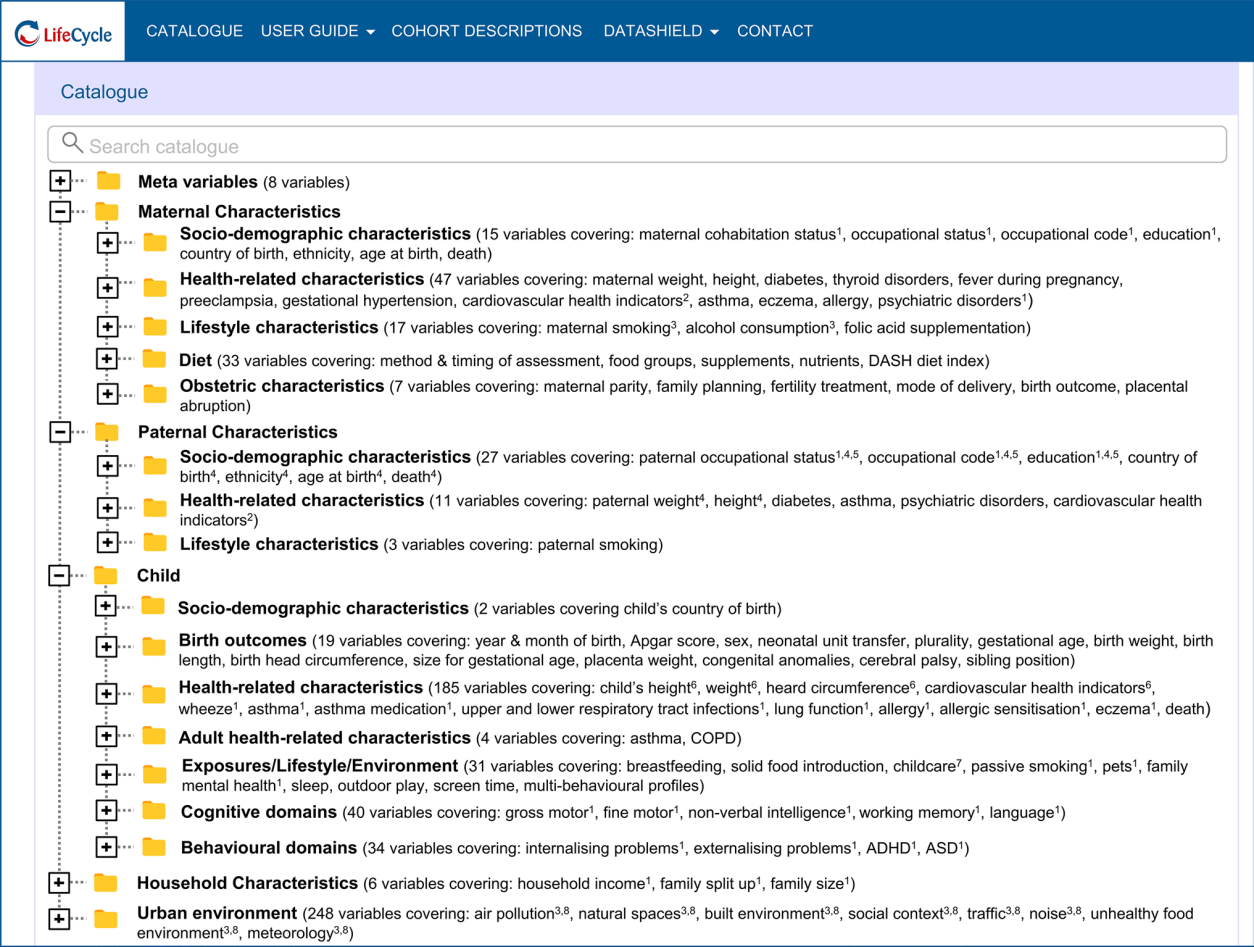
An illustration of the EU Child Cohort Network Variable Catalogue’s menu structure giving an overview of the themes included in the EU Child Cohort Network and the number of variables included in each theme. ^1^Including yearly-repeated variables with up to 18 measures between the ages of 0 and < 18 years. ^2^Including weekly-repeated variables with up to 43 measures taken between gestational weeks 0 and < 43. ^3^Including trimester-repeated variables with separate measures for the first, second and third trimesters. ^4^Including separate variables indicating the type of father the variable relates to (biological, social father, social mother, unknown). ^5^Including separate variables relating to secondary father-figures. ^6^Including monthly-repeated variables with up to 216 measures between the ages of 0 and < 216 months. ^7^Including yearly-repeated variables with up to four measures between the ages of 0 and < 4 years. ^8^Including yearly-repeated variables with up to 13 measures between the ages of 0 and < 13 years

The catalogue has been built using a logical tree structure, but variables can also be located using a search function (Fig. 3). There are plans to also incorporate descriptive summary statistics for each harmonised variable. Thus, the EU Child Cohort Network Variable Catalogue provides a comprehensive overview of the EU Child Cohort Network’s data, ensuring they are both findable and reusable, as well as contributing to the longer-term sustainability of the network.

#### Step 7b: uploading data to a data management platform for the federated analysis of data

To help ensure the sustainability and accessibility of the EU Child Cohort Network, an IT infrastructure has been implemented enabling the federated analysis of data. Full details of this infrastructure are given elsewhere [29, 56, 57]. Briefly, this infrastructure consists of secure Opal servers [58] located either at each host institution or on outsourced IT infrastructures. Once harmonisation is complete, each cohort uploads their harmonised data to their Opal server, where they remain stored, behind secure firewalls. Individual-level data are accessed via an RStudio Open Source central analysis server (https://rstudio.com/products/rstudio/#rstudio-server) using the R-based platform DataSHIELD [56], which sends blocks of code to each Opal server and then combines the summary statistics that are sent back by each Opal server. There is no transfer of individual participant data to the researcher and a number of disclosure control filters ensure analyses are non-disclosive, thus the many ethical, legal and societal implications of transferring data from one site to another are avoided.

#### Step 8: central quality-control

Quality of harmonised data was assessed at the central level by creating summary statistics for each core variable in R/DataSHIELD. This was to identify outliers and improbable values and inconsistencies in data as outlined above, but also to identify large inconsistencies between cohorts. Where large inconsistencies were found, sampling and recruitment methods and differences in the instruments used to collect data were investigated, as well as the harmonisation process itself, in order to establish to what extent these differences were real versus an artefact of differing methodology.

## Results

Table 1 provides an overview of the 17 cohorts currently contributing data to the EU Child Cohort Network. As of June 2020, the network holds data on just under 250,000 children and their parents, with contributing cohorts ranging in size from 967 to 76,569 children. This is an initial number and will increase as new cohorts and their parent-child triads join the network.

First and last year of recruitment of cohorts ranged between 1934 (HBCS) and 2016 (NINFEA) respectively. Mean age of children at recruitment ranged from -1084 days before birth (approximately -3 years, in SWS, which recruited mothers before conception) to 17 days postpartum (in CHOP). The majority of mothers enrolled in the cohorts were recruited during pregnancy (13 of the 17 currently participating cohorts).

Tables 2 and 3 summarise some key characteristics of the mother-child dyads from each cohort currently contributing data to the EU Child Cohort Network. Of note is the variation in the proportion of children born small and large for gestational age (ranging from 2.2% in CHOP to 11.2% in BiB and from 2.7% in CHOP to 14.2% in NFBC1986 for SGA and LGA respectively) and the proportion of children ever breastfed (ranging from 73.4% in EDEN to 99.6% in HBCS). Also of note is the variation in the proportion of mothers with a high level of education (ranging from 3.3% in NFBC1966, most likely reflecting the earlier year of recruitment of this cohort, to 67.5% in MoBa) and the proportion of mothers who smoked during their pregnancy (ranging from 7.6% in NINFEA which is based in Italy, where the prevalence of smoking among women and especially pregnant women is known to be lower [59], to 33.1% in Rhea). Multiparity ranged between 27% in NINFEA and 69% in NFBC1966.

Although we focus here on describing the EU Child Cohort Network’s core variables, the network also includes variables relating to the early-life exposome, encompassing both the external environment (socio-economic, migration, urban environment and lifestyle factors) and internal environment (determined from biological markers such as DNA methylation, RNA expression and metabolomics), and outcome variables relating to cardio-metabolic, respiratory and mental health. An overview of all the themes of the EU Child Cohort Network is provided in Fig. 3, together with estimates of the total number of variables included in each theme. Due to the fact that new variables are continuously being added to the network with the inception of new research projects, these numbers are highly conservative.

The core variables consist of a set of 130 basic, principally lowest common denominator variables, available in the majority of participating cohorts and required for many analyses within the scope of LifeCycle and other lifecourse epidemiology research themes. Of these, seven are so-called “meta variables”, consisting of mother, child, pregnancy, and cohort identifiers, and variables providing the age of recruitment and country of cohort. The remaining variables consist of 96 non-repeated variables and 17 yearly-repeated variables with up to 18 measures between the ages of 0 and < 18 years, together capturing maternal, paternal and child health, lifestyle, socio-demographic characteristics, mother’s obstetric history, birth outcomes and household exposures. There are also two trimester-repeated variables capturing maternal smoking and alcohol consumption during pregnancy, four yearly-repeated variables with up to four measures between the ages of 0 and < 4 years capturing childcare and four monthly-repeated variables with up to 216 height or weight measures between the ages of 0 and 215 months. The full list of EU Child Cohort Network core variables is provided in Online Resource 1 and also in the EU Child Cohort Network Variable Catalogue (http://catalogue.lifecycle-project.eu). Since the EU Child Cohort Network Variable Catalogue is dynamic and regularly expanded with both new variables and newly participating cohorts, the statistics reported there may differ from what is presented here.

**Fig. 4:**
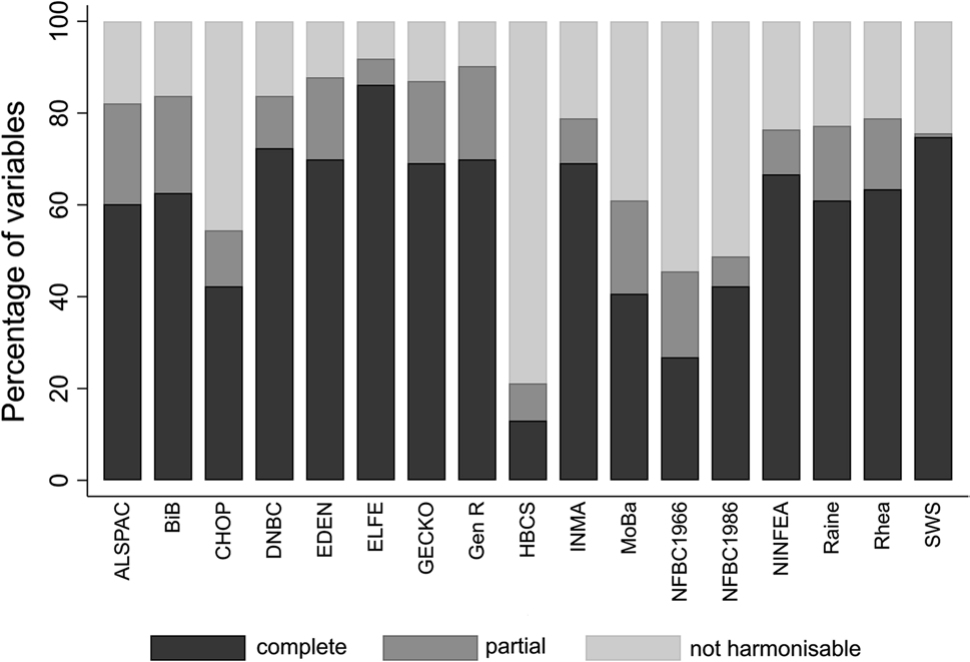
Percentage of EU Child Cohort Network core variables harmonised by each cohort. The figure displays the percentage of the 123 core variables listed in Online Resource 1 (excluding meta-variables) harmonised by each cohort. Shading of bars displays the degree of matching within each cohort: black bars represent percentage of completely harmonised variables; dark grey bars represent percentage of partially harmonised variables; light grey bars represent percentage of variables that were not harmonizable (impossible harmonisation)

Excluding the seven meta-variables, the percentage of core variables harmonised by cohorts ranged from 21% for HBCS to 92% for ELFE (Fig. 4). Missing variables are due to cohorts not having the data required to harmonise the variable. Twelve of the 17 cohorts currently included in the EU Child Cohort Network were able to harmonise at least 50% of core variables completely, and 12 of the 17 cohorts were able to harmonise at least 75% of core variables either completely or partially.

**Fig. 5:**
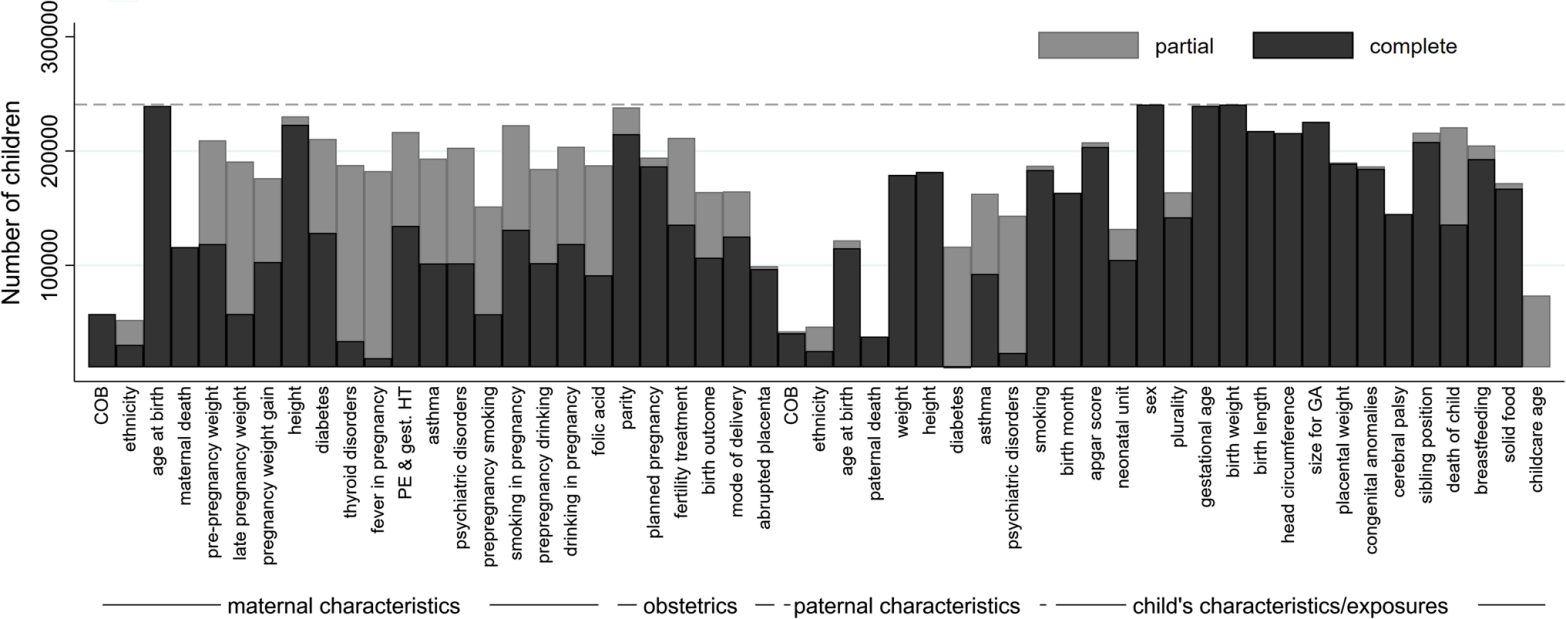
Harmonised non-repeated core variables in the EU Child Cohort Network. Bars display the number of children with either a partially (grey bars) or completely (black bars) harmonised core variable for each of the main themes/exposures. The dashed line represents the total number of children (240,684), as of June 2020, contributing data to the EU Child Cohort Network with all three of the following variables harmonised: (1) birth weight, (2) sex, (3) at least one height or weight measurement taken at ≥ 1 year. *COB* country of birth, *PE* pre-eclampsia, *gest. HT* gestational hypertension, *size for GA* size for gestational age

**Fig. 6:**
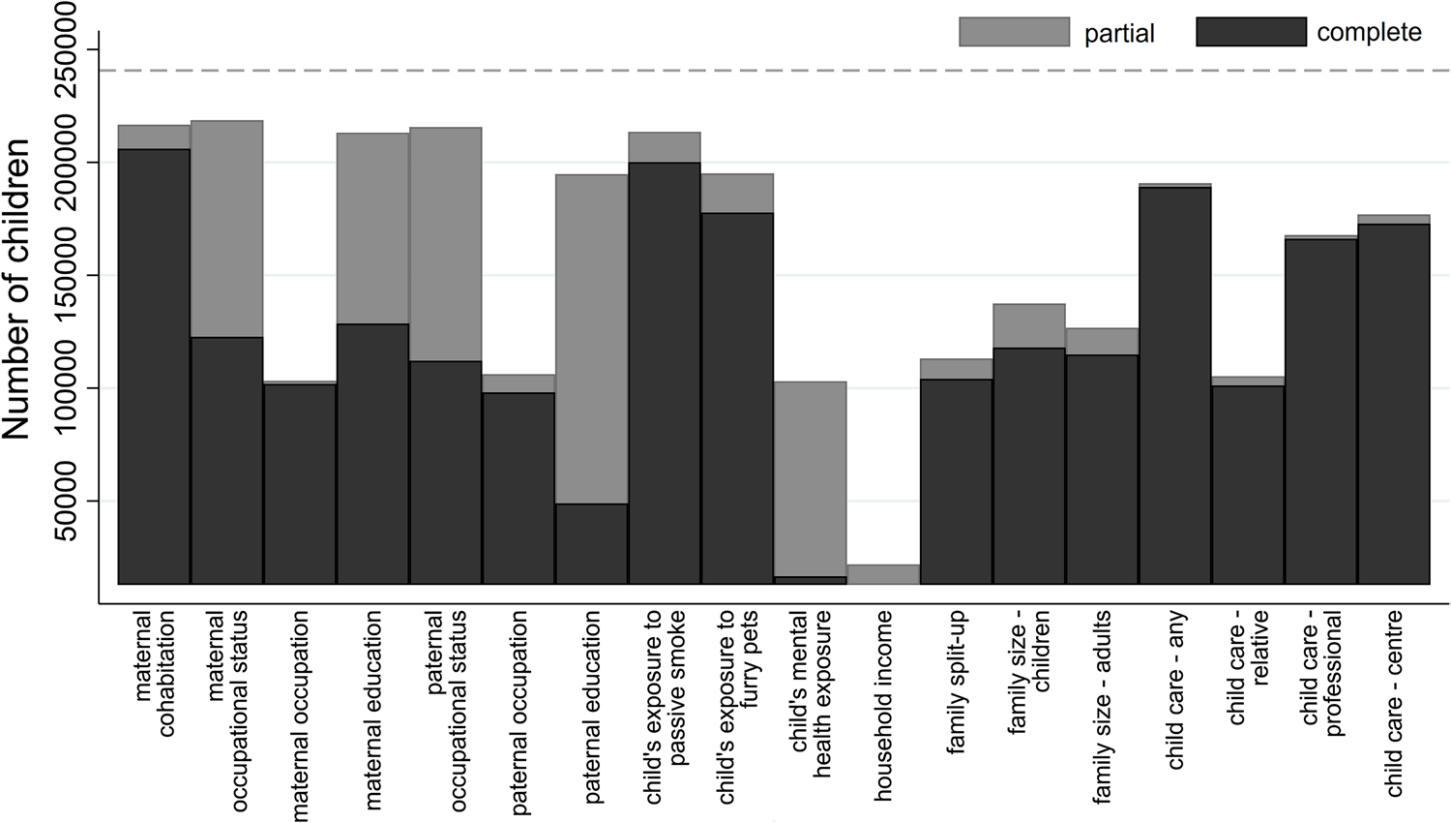
Number of children in the EU Child Cohort Network with yearly-repeated measure core variables. Bars display the number of children with at least one measure between the ages of zero and three (child-care variables) or zero and seventeen (all other variables), either partially (grey bars) or completely (black bars) harmonised. The dashed line represents the total number of children (240,684), as of June 2020, contributing data to the EU Child Cohort Network with all three of the following variables harmonised: i) birth weight, ii) sex, iii) at least one height or weight measurement taken at ≥ 1 year

**Fig. 7:**
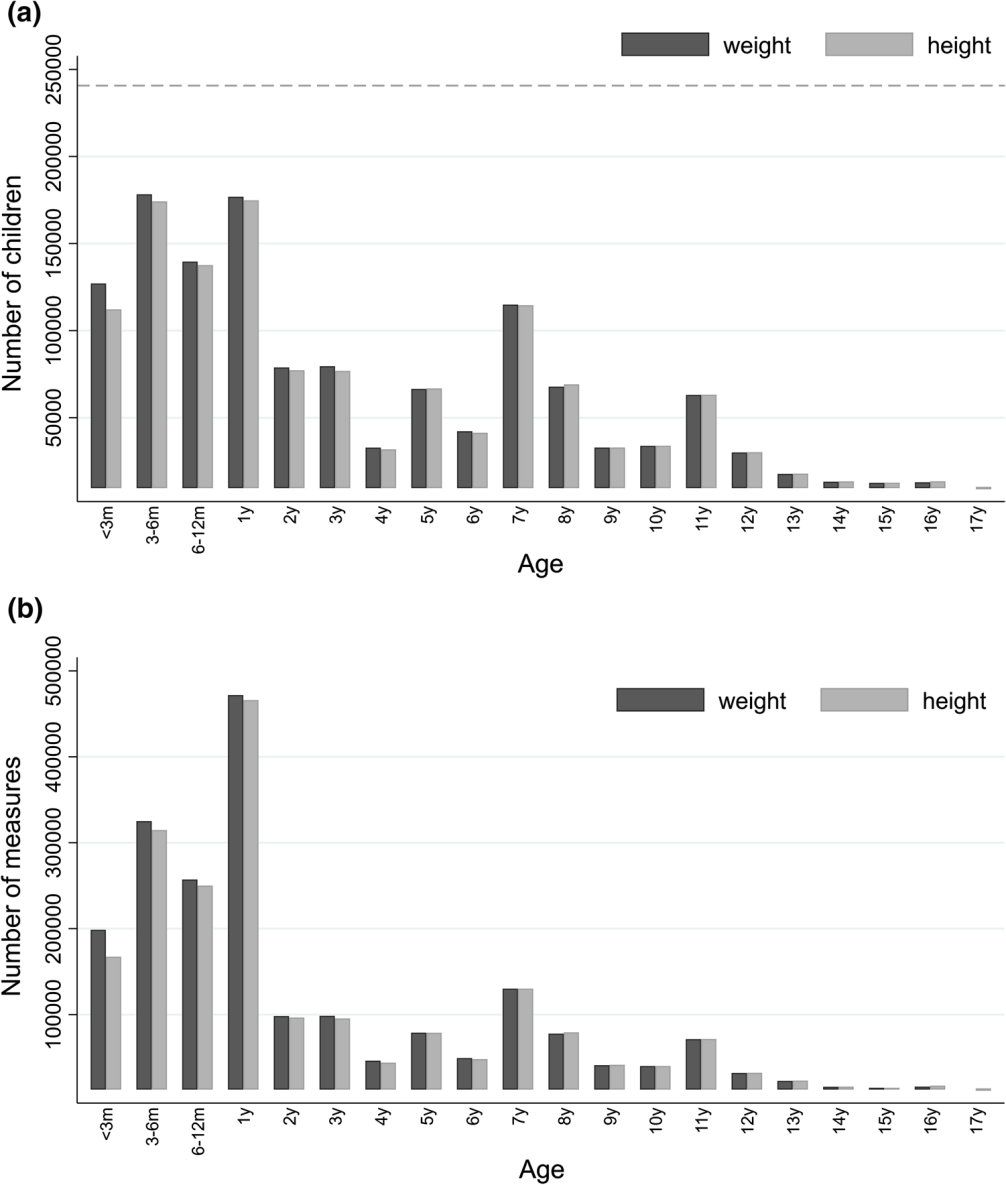
Weight and height data in the EU Child Cohort Network. Graphs display **a** number of children in the network with at least one weight (dark grey bars) or height (light grey bars) measure at < 3 months, 3–6 months, 6–12 months and yearly intervals from 1 to 17 years; **b** total number of weight (dark grey bars) and height (light grey bars) within each age band (i.e. one child may contribute multiple measurements within each age band)

Figures 5, 6 and 7 give an overview of the number of EU Child Cohort Network children (i.e. from all cohorts combined) with harmonised core data. Of the non-repeated core variables (Fig. 5), themes with the most complete data are those relating to maternal characteristics (specifically, age at birth, height, smoking during pregnancy, parity) and child-related characteristics (specifically, sex, gestational age at birth, birth weight, birth length, size for gestational age and death of the child), with more than 217,000 children as of June 2020 having harmonised data relating to these exposures. Notably fewer children have data relating to mother and father’s country of birth and ethnic background, perhaps due to their sensitive nature [60].

An overview of the number of EU Child Cohort Network children with harmonised yearly-repeated core variables, which allow for time-varying exposure statuses, is displayed in Fig. 6. Over 80% of children in the network have at least one harmonised measure of cohabitation status, mother’s occupational status, mother’s education level, father’s occupational status, father’s education level, and child’s exposure to pets and cigarette smoke, whilst relatively few children (< 10%) have harmonised data on household income. For growth data (Fig. 7), the greatest density of measures in the network is between the ages of 0 and < 1 year, with a total of 780,993 and 732,202 weight and height measurements available between these ages respectively, an average of three weight and height measures per child. Large amounts of growth data are also available for ages 1– < 2 years and 7– < 8 years, with over 72% and 47% of children having harmonised weight and height data at these ages respectively, whilst relatively few children currently have weight and height data from 14 years and onwards, partly because many cohorts have not yet reached that age.

## Discussion

The Horizon 2020 LifeCycle Project is a collaboration between scientists from more than 17 pregnancy and birth cohorts from across Europe and Australia. It builds upon the expertise gained from previous collaborations such as the CHICOS, Enrieco and BioSHARE projects in order to establish an open and sustainable data resource known as the EU Child Cohort Network so as to facilitate research on the influence of early-life stressors on later health outcomes.

Here we have described the EU Child Cohort Network, focussing on its core variables, including the protocol developed to harmonise these data and thus make them interoperable. We have also described the EU Child Cohort Network Variable Catalogue, developed to ensure that these and other data in the network are both findable and re-usable. These data will be analysed using a federated analysis platform, meaning there is no need to physically transfer data, and so data are ultimately more accessible to the researcher.

As well as the harmonised core data described here, the EU Child Cohort Network also contains data relating to the early-life exposome, and repeated measures of cardio-metabolic, respiratory and mental health. An additional feature of the network is the varied social, cultural and political environments of the cohorts. Thus, the EU Child Cohort Network constitutes an invaluable data resource, not only in terms of the number of participants included, but also in terms of its breadth, depth and diversity. This will ultimately enable the application of a range of analytical approaches to help infer causality, and identify possible target groups for improved cardio-metabolic, respiratory and mental health across the lifecourse.

However, the creation of such a data resource is not without its limitations. Firstly, the resources required to create a common dataset, i.e. harmonise data, should not be underestimated. Harmonising data is difficult, time consuming and requires considerable investment by all involved. Although central harmonisation, whereby individual participant data are sent to one coordinating centre which harmonises all variables, is often viewed as the more optimal approach, this is not without its drawbacks. Firstly, there are many ethico-legal challenges surrounding the transfer of data; secondly, it takes considerable investment by the data manager to become acquainted with a cohort’s data, scaled up 17 times in the case of the EU-Child Cohort Network, potentially leading to errors. It is for these reasons, and the fact that the EU Child Cohort Network is an open network, such that new cohorts are invited to join and are continually joining, that LifeCycle opted for local harmonisation. Here, harmonisation is carried out locally by each cohort, coordinated by a central coordinating centre. This of course has the limitation that harmonisation protocols may be interpreted differently by different cohorts. We have tried to limit this possibility in LifeCycle by providing detailed instructions and maintaining regular contact with data managers. We have also implemented a number of data quality checks, applied both locally and centrally. These include checks to ensure that harmonised variables match those detailed in the harmonisation manual and to identify outliers or improbable values, or any inconsistencies in measures within or between cohorts. Good documentation of all harmonisation steps is key to diagnosing any inconsistencies, which we have ensured in LifeCycle by establishing the EU Child Cohort Network Variable Catalogue.

Another drawback of data harmonisation is that the end product is often the “lowest common denominator”. For any given variable, some cohorts will inevitably have more detailed variables than other cohorts. In an attempt to create a common variable achievable by all cohorts, more detailed variables are stripped down to simpler versions, inevitably resulting in some loss of information. This may also involve deciding that in some cohorts there is insufficient data to harmonise a variable. Harmonisation is thus a balancing act between retaining as much information as possible while ensuring data are fully comparable [61].

So, if the creation of a common dataset is such a tremendous task and the end product may, in some instances, be less detailed than the original data, why bother? Increased statistical power is one obvious advantage. Combining data from several cohorts to increase power allows rarer, but equally important and often more devastating [62], diseases and rare determinants to be studied. Larger sample sizes also allow for more powerful statistical analyses, such as exploring multiple interactions, complex nonlinear relationships, small effects or dose responses [63]. While national registers offer the possibility of creating birth cohorts of an order of magnitude larger than the EU Child Cohort (for e.g. Nordic register-based cohort studies [64, 65]), these typically lack the in-depth lifestyle and behavioural data obtained from questionnaires, or physiological data obtained from detailed clinical examinations. National register data are in addition likely to offer less diversity with respect to social, cultural and political environment. Cross-cohort collaborations also allow fine resolution biological data to be shared, such as medical images or metagenomic data, that may be prohibitively costly to obtain from the entire cohort and therefore only collected from a sub-population of the cohort.

A larger sample size is not the only benefit of cross-cohort collaborations. Combining data also offers the opportunity to study populations typically under-represented in cohort studies, for example individuals from lower socio-economic backgrounds or ethnic minority groups. Heterogeneities between cohorts can be utilised to strengthen causal inference. For example, differing confounding structures allows the untangling of true associations, whilst replication of findings across different populations with differing gene pools, and cultural and socio-economic structures, helps to rule out chance findings while also establishing the generalisability of results. Geographical, intergenerational and period effects can also be examined to find new associations and generate new hypotheses.

While it could be argued that an easier and potentially less time-consuming approach to combining data from several studies is the more conventional systematic review and meta-analysis of published data, this has a number of disadvantages compared to individual participant data (IPD) meta-analysis. Published data are often subject to selective reporting and publication bias, lack harmonised measures, and offer limited scope and flexibility in terms of statistical analysis, and few opportunities, if any, for data checking [66, 67].

The added value that the collaboration itself brings should also be highlighted: the opportunity to share ideas and methodology, learn from each other, and ultimately strengthen research outputs. Also the increased use of data and exchange opportunities for researchers. Scientific collaboration also facilitates the dissemination of both results and ideas/hypotheses, as well as creating opportunities for interdisciplinary research.

In conclusion, the EU Child Cohort Network offers an invaluable data resource for studying how early-life exposures influence health trajectories throughout the lifecourse. This is both in terms of the number of its participants, and the breadth and depth of its data. Here we share the approach taken within LifeCycle to harmonise the network’s core data and describe the EU Child Cohort Network Variable Catalogue established to ensure that the network’s data are both findable and reusable. We also highlight some of the great benefits of cross-cohort collaboration. Having hopefully convinced the reader of the benefits of the EU Child Cohort Network and similar cross-cohort collaborations, we end with a plea to other cohorts to join the network and share their data, and to researchers to utilise this incredible resource. Both cohorts and researchers can join the network by contacting lifecycle@erasmusmc.nl.

## Supplementary Information

Below is the link to the electronic supplementary material.

Supplementary material 1 (XLSX 34 kb)

Supplementary material 2 (DOCX 40 kb)

## Data Availability

Proposals for research based on EU Child Cohort Network data can be put forward by contacting the coordinating centre (lifecycle@erasmusmc.nl).

## Code availability

Available on the LifeCycle GitHub (https://github.com/lifecycle-project).
